# Sacrificial Dissolution of Zinc Electroplated and Cold Galvanized Coated Steel in Saline and Soil Environments: A Comparison

**DOI:** 10.3390/ma14040744

**Published:** 2021-02-05

**Authors:** Ameeq Farooq, Umer Masood Chaudry, Ahsan Saleem, Kashif Mairaj Deen, Kotiba Hamad, Rafiq Ahmad

**Affiliations:** 1 Corrosion Control Research Cell, Department of Metallurgy and Materials Engineering, University of the Punjab, Lahore 54590, Pakistan; ameeq.farooq@gmail.com (A.F.); ahsansaleem318@gmail.com (A.S.); 2 School of Advanced Materials Science & Engineering, Sungkyunkwan University, Suwon 16419, Korea; umer@skku.edu; 3 Department of Materials Engineering, University of British Columbia, Vancouver, BC V6T 1Z4, Canada

**Keywords:** zinc rich coatings, electrochemical impedance spectroscopy, sacrificial coatings

## Abstract

To protect steel structures, zinc coatings are mostly used as a sacrificial barrier. This research aims to estimate the dissolution tendency of the electroplated and zinc-rich cold galvanized (ZRCG) coatings of a controlled thickness (35 ± 1 μm) applied via brush and dip coating methods on the mild steel. To assess the corrosion behavior of these coated samples in 3.5% NaCl and 10% NaCl containing soil solutions, open circuit potential (OCP), cyclic polarization (CP), and electrochemical impedance spectroscopy (EIS) tests were performed. The more negative OCP and appreciably large corrosion rate of the electroplated and ZRCG coated samples in 3.5% NaCl solution highlighted the preferential dissolution of Zn coatings. However, in saline soil solution, the relatively positive OCP (>−850 mV vs. Cu/CuSO_4_) and lower corrosion rate of the electroplated and ZRCG coatings compared to the uncoated steel sample indicated their incapacity to protect the steel substrate. The CP scans of the zinc electroplated samples showed a positive hysteresis loop after 24 h of exposure in 3.5% NaCl and saline soil solutions attributing to the localized dissolution of the coating. Similarly, the appreciable decrease in the charge transfer resistance of the electroplated samples after 24 h of exposure corresponded to their accelerated dissolution. Compared to the localized dissolution of the electroplated and brush-coated samples, the dip-coated ZRCG samples exhibited uniform dissolution during the extended exposure (500 h) salt spray test.

## 1. Introduction

Coatings are widely used to protect metals from corrosion, but their performance depends on how much they physically restrict the approach of water and oxygen to the coating/substrate interface [1]. Coating experts say that even an excellent coating has internal defects and that any localized mechanical damage of the coating may deteriorate the integrity of the structure [2]. For the protection of a steel structure, the application of coatings has a dual character, i.e., sacrificial nature and barrier characteristics are considered important. Zinc-based galvanic coatings can fulfill the requirement by providing good sacrificial protection in the initial stage of service life. When the zinc-based coatings are exposed to aqueous solutions for an extended period, these show good barrier characteristics due to the formation of insoluble species on the surface that are alkaline, e.g., Zn(OH)_2_ [3]. However, under an acidic and saline environment, these coating may deteriorate rapidly and their protection capability is limited. 

Zinc metal pigment was widely used in such coatings due to its sacrificial behavior, which protects steel structures, i.e., bridges, marine, and oil platforms. The overall anodic behavior of Zn-based coatings could cathodically protect the local defects and limit the aggressive corrosion of the steel substrate [4,5,6,7,8,9,10,11]. In soil, the highly inhomogeneous environment shows a wide range of physical and chemical properties that depend on the geological and environmental conditions [12]. The process of zinc coating application on the steel surface is also a challenging issue for the industry. For instance, the hot galvanizing of steel requires a large setup, delicate operational controls, and optimized conditions. On the other hand, electro-galvanizing or Zn-electroplating on the steel surface is another well-established process [13]. However, the active nature of the electroplated Zn coating and its rapid dissolution limit its protection capacity, which is considered the main obstacle in its wide applicability. To avoid the accelerated dissolution of the electroplated Zn, many commercial post-treatment procedures, i.e., application of conversion coatings, are in use [14]. These treatments involve the use of many toxic species, such as hexavalent chromium (Cr^6+^), which is a real environmental concern. The use of molybdates, permanganates, and tungstate species in the post-chemical treatments or the application of conductive polymeric films on the surface of electroplated Zn have also been reported in the literature [15,16,17,18]. Here, it can be evaluated that pre-cleaning of the steel substrate in highly acidic and/or alkaline solutions and application of post-treatment procedures is a huge concern for the environment and general public health. To address these issues, the application of the cold galvanizing coating is considered one of the suitable options, as it uses environment-friendly materials. Cold galvanizing has great flexibility and can be applied to many intricated and in-service structures. In other words, the cold-galvanized coatings can be easily applied to complex structures without dismantling the parts if a repair is required [19]. Shreepathi et al. [20] studied the self-healing properties of the zinc-based organic coating and concluded that the sacrificial efficiency of zinc-rich pigment coating depends on the quantity and purity of the zinc particles in the polymeric matrix. The zinc particle size plays a vital role in the performance of zinc-rich paints. The mixing of various size zinc particles could improve the electrical conductivity between the particles in addition to the increase in corrosion resistance of the Zn-rich coatings [21,22]. It has been evaluated that the metallic Zn coatings produced via hot/electro-galvanizing dissolve in an uncontrolled manner and require post-chemical treatments. The chemical conversion layers or application of the conducting polymeric films on these Zn coatings may adversely affect their functionality and could increase the operational cost of the coating in addition to posing a threat to the environment.

As discussed, the Zn electro-galvanized coating requires the application of an additional conversion layer or a polymeric film for controlling its dissolution tendency. On the other hand, application of cold-galvanizing is a single step and does not require any post-treatment procedure, and it presents an acceptable dissolution rate. Based on these reasons, in this study, the desired function of the cold-galvanized (polymer-based zinc-rich) coatings, i.e., sacrificial dissolution tendency and protection capability, is evaluated. These coatings were applied via brushing or dipping, and their dissolution tendency is measured under the same conditions. A comparison between these composite (Zn-rich polymer matrix) coatings and electroplated metallic Zn (without post-treatment) is also given to estimate their dissolution behavior and effectiveness to protect steel substrate from corrosion. In this regard, the open-circuit potential (OCP), cyclic polarization (CP), and electrochemical impedance spectroscopy (EIS) tests of the steel substrate, cold-galvanized and electro-galvanized (Zn electroplated) coatings were performed in saline and soil environments. 

## 2. Experimental Technique

The mild steel panels were cut from a 2 mm thick sheet into 15 cm × 7.5 cm dimensions. The chemical composition of mild steel was 0.19 wt.% C, 0.22 wt.% Si, 0.4 wt.% Mn, 0.073 wt.% Cr, and Fe balance. The surface contamination and corrosion product of the steel panels were removed by using a rotating wire brush. The steel panels were degreased ultrasonically in ethanol solution and dried in air. The surface roughness was measured by a roughness profilometer (Mitutoyo SJ-201, Mitutoyo, Kawasaki, Japan). The average roughness (R_a_) was measured to be 4.12 ± 0.5 μm. The zinc electroplated coating was applied galvanostatically (at 0.5 A·dm^−2^) by immersing the steel panels for 30 min in a solution (pH 4.5) containing zinc chloride 60 g·L^−1^, potassium chloride 200 g·L^−1^, boric acid 30 g·L^−1^, and brightener 10 mL·L^−1^. The pure zinc (99.9%) was used as a counter electrode (anode) in a two-electrode cell, and the solution temperature was maintained constant at 40 °C. 

The Zn rich (96% metallic Zn) mixed in acrylic-based resin and xylene (as a solvent) paint (Roval ^®^, Osaka, Japan) was applied to the steel samples by brushing and dip coating methods. This Zn-rich cold galvanized (ZRCG) coating was applied on mild steel panels by using a dip coating machine. The steel panels were vertically dipped in the paint for 15 s and pulled gradually at approximately 1 cm-min^−1^ to produce uniform thickness coatings. A few mild steel panels were also brush coated by applying the ZRCG coatings. It was difficult to achieve the uniform and homogeneous coating by manual brush coating. However, the samples that had the same coating thickness (as galvanized and dip coatings) were selected in the study. The ZRCG coated panels were dried in a closed chamber with a controlled humidity (60%) level of 25 °C after 3 h. Table 1 shows the designation of the uncoated and coated panels. The dry coating thickness was measured using a coating thickness meter (456 Elcometer, Elcometer, Manchester, UK). The surface roughness was also measured after the drying of the coated samples. The average roughness (*R*_a_) of mild steel and coated panels is illustrated in Table S1. The adhesion strength of all coated samples was rated through the tape test according to ASTM D3359. All coatings qualified in the adhesion test and were rated/classified as 5B.

All the samples were designated as mild steel (MS), Zinc Electroplated (GS), ZRCG coating applied via brushing (BS), and ZRCG coating applied via a dipping method (DS), as shown in Table 1. 

The electrochemical behavior of the MS, GS, BS, and DS coated samples was determined by exposing in 3.5% NaCl and saline soil solutions. The 3.5 wt.% NaCl solution was prepared by dissolving the desired amount of NaCl in DI-water and by adjusting the final pH of 7.86 with the addition of NaOH. The chemical composition of the soil used in electrochemical testing is illustrated in Table S2. The soil containing 10 wt.% NaCl solution with a pH of 8.01 was also prepared to determine the electrochemical behavior and protection capability of the MS and coated samples. Electrochemical tests of the uncoated and coated steel samples were conducted in a paint cell having an exposed surface area of 9.06 cm^2^ and coupled with a Potentiostat (1000E, Gamry, Warminster, PA, USA). The schematic diagram of the electrochemical cell is illustrated in Figure S1. In this cell, the graphite rod was used as a counter electrode. Ag/AgCl electrode (199 mV vs. standard hydrogen electrode (SHE)) and Cu/CuSO_4_ (318 mV vs. SHE) were used as reference electrodes in 3.5% NaCl and saline soil solution, respectively. 

The OCP of the MS, electroplated, and ZRCG coated samples was measured after one hour and 24 h of immersion in both test solutions. The impedance spectra of these samples were also obtained after one hour and 24 h of immersion in both solutions within a frequency range of 100 kHz–10 mHz. A small alternating potential amplitude of ±5 mV_rms_ vs. OCP was applied at direct current (DC) potential of 0 V vs. OCP. Cyclic polarization scans were performed after 24 h of exposure to both electrolytes within the potential range of −0.3 mV vs. OCP to apex potential of 1.0 V vs. OCP with a forward scan rate of 2 mV·s^−1^. The final potential of −0.3 mV vs. OCP was selected in the reverse scan to estimate the difference in the current output. The electrochemical tests were conducted in triplicate to ensure reproducibility and to determine the variation in the results. Salt spray tests of the coated panels were also performed according to ASTM B-117 standard test protocol under 85% relative humidity in 5 wt.% NaCl solution at 35 °C. The pH of this solution was adjusted to 6.85. Before placing the coated samples in the salt spray chamber, one side of the coated sample was cross-hatched to estimate the protection capability and localized dissolution tendency of the coated sample. The coated panels were exposed in a salt spray chamber for 500 h at an angle of 30° parallel to the flow direction of the saline fog. 

## 3. Results and Discussion

### 3.1. Coating Morphology and Thickness and Roughness Measurement

**Figure 1:**
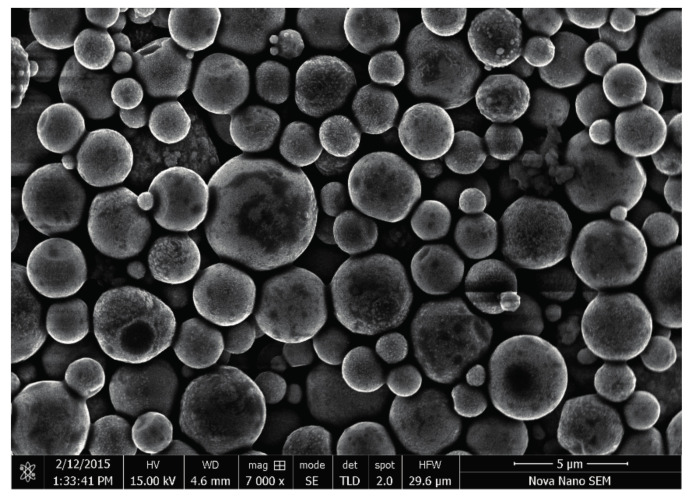
Dispersion of Zn particles in the acrylic resin showing the large inter-particulate contact.

Figure 1 shows the scanning electron micrograph of the acrylic-based Zn-rich Roval ^®^ coating. The cold galvanized compound paint contains zinc particles of varying sizes. The round shape zinc particle diameter varies from 0.1 μm to 5 μm. During the initial stage, the protection of steel substrate by cold galvanized coating can be enhanced by improving the electrical contact between the zinc particles [21], which can be achieved by adding zinc particles of different sizes to the resin. The large concentration of small size zinc particles homogeneously dispersed in the acrylic resin would occupy the space between the large zinc particles and may facilitate the inter-particulate contact as shown in Figure 1.

The average dry coating thickness of 35 ± 1 μm of the electroplated and cold galvanized coating was obtained by optimizing the coating application conditions. However, compared to the DS sample, a relatively large variation in the coating thickness (approx. ± 10%) was observed in the case of the BS sample, possibly due to the variation in the manually applied coating procedure. The average roughness (*R*_a_) of the zinc electroplated sample was 0.45 μm whereas dipped and brushed cold galvanized samples have presented average roughness of 0.80 and 1.15 μm, respectively. The relatively large surface roughness of the cold galvanized coating applied via brushing was related to the inhomogeneity in the manually applied coating. The electroplating of zinc on the surface produced a refined grain structure and resulted in a smooth surface. The relatively higher roughness of the dip-coated sample compared to the electroplated one was possibly related to the micro-sized dispersed Zn particles in the acrylic resin. But the machined controlled dipping and removal of the steel panels (DS samples) could produce a smooth and homogenous surface and resulted in relatively low surface roughness compared to the BS samples.

### 3.2. Open Circuit Potential 

**Figure 2:**
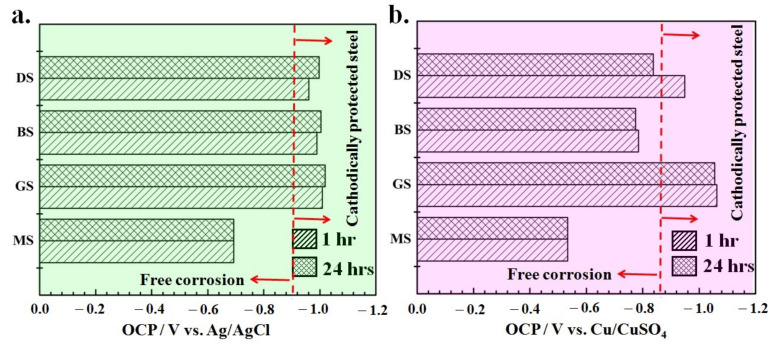
Comparison of the open circuit potential in (**a**) 3.5% NaCl and (**b**) saline soil. The open-circuit potential (OCP) values were measured after 1 and 24 h of exposure.

The OCP of the electroplated and cold galvanized steel panels was determined to estimate the electrochemical potential of the surface and its tendency to react with the electrolyte [23,24,25]. Figure 2 shows the OCP of the MS, GS, BS, and DS samples after one and 24 h of immersion in 3.5% NaCl and saline soil. In both environments, the OCP of the coated panels was shifted to more active potential (more negative) than the bare (MS) sample, which shows the sacrificial nature of the coating and its tendency to protect the steel substrate. According to the National Association of Corrosion Engineers (NACE) cathodic protection criteria for the steel structures, the potential of −900 mV vs. Ag/AgCl and −850 mV vs. Cu/CuSO_4_ in seawater and soil environments, respectively, is recommended [26]. As shown schematically in Figure 2, the OCP of the electroplated and ZRCG coated samples in both saline and soil conditions lies in the cathodic protection limit (except for the BS sample in the saline soil solution).

In 3.5% NaCl solution, the OCP of the MS sample was −693 mV vs. Ag/AgCl, whereas a more negative OCP of the GS (−1009 mV), BS (−989 mV) and DS (−961 mV) vs. Ag/AgCl samples was observed after one-hour immersion. The appreciably negative OCP of the electroplated and ZRCG coated samples compared to MS corresponded to the active state of the surface coating relative to the substrate. This indicated the preferential dissolution of the surface coatings compared to the steel substrate. In other words, the sacrificial dissolution of the coating prevents the steel substrate from corroding if any surface damage occurs to the coating and the substrate is locally exposed to the seawater. It is believed that effective sacrificial coatings must have a small potential difference between substrate and coatings. If there is a large difference in the OCP, sacrificial coating may dissolve quickly, and the substrate is exposed to the aggressive environment. Moreover, there is a possibility of hydrogen (H_2_) evolution, and its absorption into the steel substrate may lead to hydrogen embrittlement of the steel structure [27,28]. Figure 2a shows a maximum potential difference of −316 mV vs. Ag/AgCl between MS and GS samples, which may correspond to fast coating dissolution. On the other hand, the DS sample presented a relatively small potential difference of −268 mV vs. Ag/AgCl compared to the BS sample (−296 mV vs. Ag/AgCl). No significant difference in the OCP of MS and coated panels was observed after 24 h of exposure, which highlighted their improved stability and tendency to dissolute uniformly.

Figure 2b shows the OCP of the MS and coated panels in a soil environment after one hour and 24 h of exposure. The OCP of MS was −535 mV vs. Cu/CuSO_4_, whereas the GS, BS, and DS samples present OCP of −1061, −784, and −951 mV vs. Cu/CuSO_4_, respectively, after a one-hour exposure to saline soil. The maximum potential difference between GS and MS was found to be about −526 mV vs. Cu/CuSO_4_, whereas BS and DS presented a relatively small potential difference of −249 and −416 mV vs. Cu/CuSO_4_, respectively. However, the OCP of the BS samples was lower than the protection criteria (−850 mV vs. Cu/CuSO_4_) according to NACE standards [26]. No significant change in the OCP of MS, GS, and BS was observed in saline soil; however, the OCP of the DS sample shifted to less negative (−810 mV vs. Cu/CuSO_4_) after 24 h, as shown in Figure 2b.

### 3.3. Electrochemical Impedance Spectroscopy

**Figure 3:**
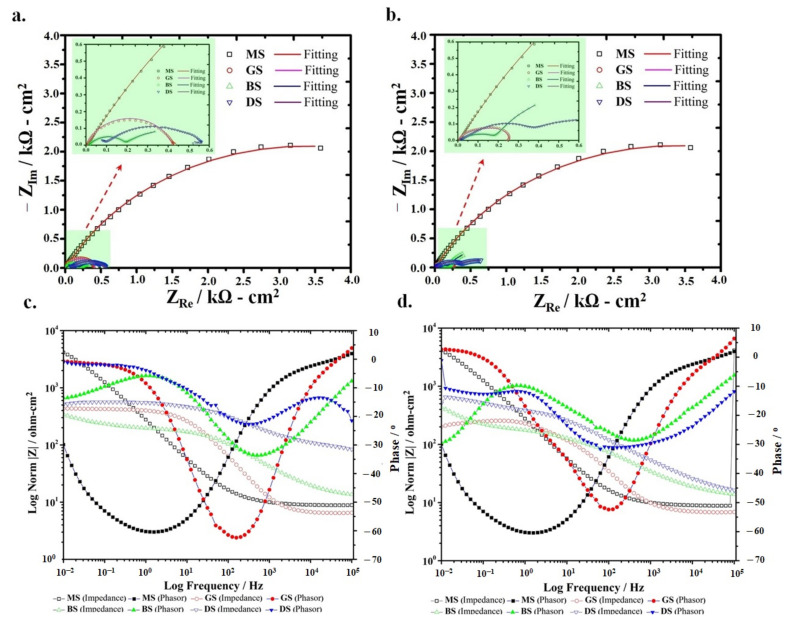
Mild steel (MS)and Zn-coated samples in 3.5% NaCl Nyquist plots of after (**a**) one hour and (**b**) 24 h of exposure and Bode plots after (**c**) one hour and (**d**) 24 h of exposure.

**Figure 4:**
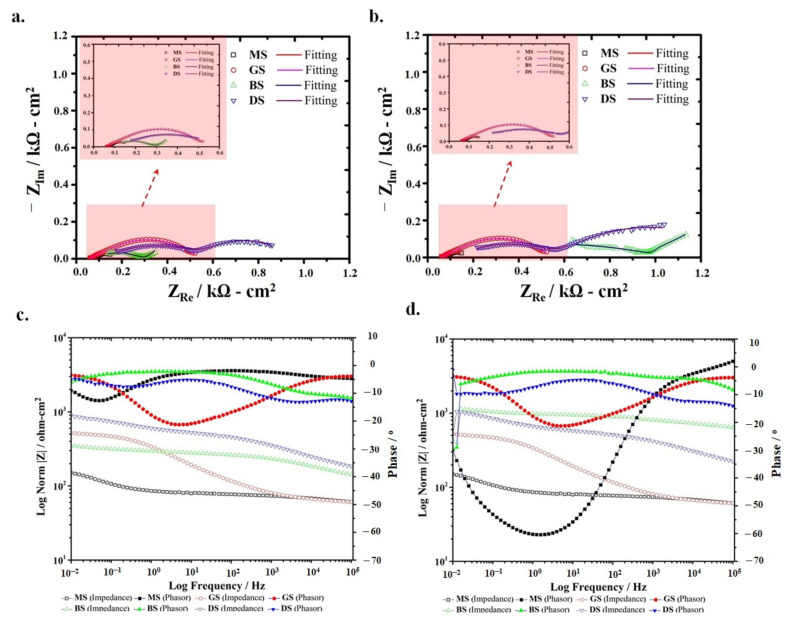
MS and Zn-coated samples in Soil Nyquist plots of after (**a**) one hour and (**b**) 24 h of exposure and Bode plots after (**c**) one hour and (**d**) 24 h of exposure.

**Figure 5:**
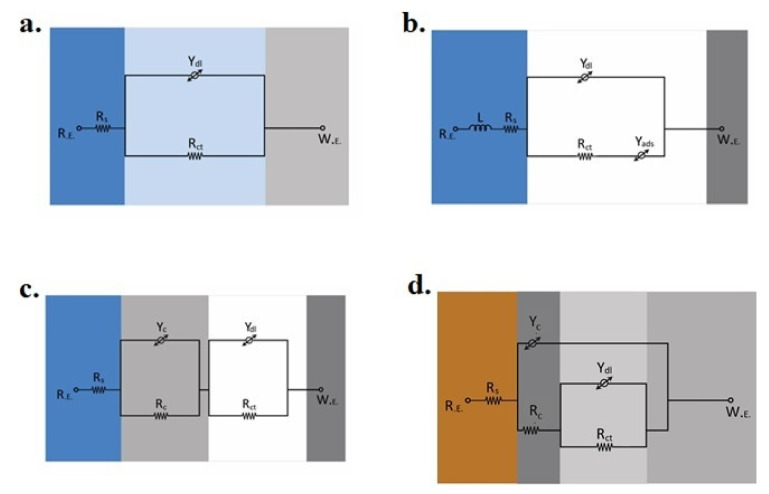
Equivalent electrical circuit models (**a**) Randle model, (**b**) effect of adsorption simulated by adding adsoption element (*Y*_ads_) in Randle circuit, (**c**) two time-constants in series, and (**d**) two time-constants in parallel combination were used to fit the experimental impedance spectra of MS and Zn electroplated and zinc-rich cold galvanized (ZRCG) coated samples.

To investigate the in-situ dissolution behavior of coated samples in both 3.5% NaCl and soil environments, the EIS analysis was carried out by applying a small alternating current (AC) potential amplitude (5 mV) at 0 V DC potential versus OCP. Figure 3 and Figure 4 show the Nyquist plots of uncoated and coated samples in the saline and soil environments after one and 24 h of immersion, respectively. The quantitative information was obtained by simulating the impedance spectra to the electrical equivalent circuit (EEC) models as shown in Figure 5. The experimental spectra were fitted to the theoretical model impedance trends by using estimated initial values (evaluated from the Nyquist plots) in the calculations. In these EEC models, *R*_s_ is the electrolyte resistance (due to the ions present in the 3.5% NaCl and soil). *R*_c_ and *Y*_c_ represent the coating resistance and non-ideal coating capacitance (constant phase element), respectively. The charge transfer resistance (exhibiting the kinetics of electrochemical processes at the substrate/electrolyte interface) and constant phase element associated with the surficial distribution of the electrical double layer (developed on the active sites) are indicated as *R*_ct_ and *Y*_dl_, respectively. At high frequency, the origin of inductance, L (positive imaginary impedance component, *Z*_img_) could be associated with the cell design and current induced in the cell cable. The Warburg impedance (W) is related to the diffusion of ionic species through the coatings, and *Y*_ad_ is the constant phase element associated with the localized absorption of the ionic species at the active sites on the coatings [29]. The quantitative information of the impedance parameters was obtained by fitting the EEC models to the experimental impedance spectra as given in Table 2.

Figure 3a shows the one time constant (a single time constant) impedance spectrum of the MS sample after one-hour immersion in 3.5% NaCl solution, which is related to its dissolution behavior. Compared to the impedance trend obtained after one hour, no noticeable difference in the impedance trend of the MS sample was observed after 24 h of immersion, which indicated the unchanged dissolution behavior of the mild steel. 

As shown in Figure 3a, the relatively small semi-circle of the coated steel samples (GS, BS, and DS) immersed in a 3.5% NaCl solution corresponded to the accelerated dissolution of the sacrificial Zn coatings. In other words, the large diameter of the capacitive loop of the MS sample compared to the GS, BS, and DS indicated that the Zn sacrificial coatings could dissolve faster than the MS sample. Qualitatively, these spectra also validated that the applied Zn coatings could cathodically protect the MS sample. The diameter of the semi-circle of Nyquist plots of GS, BS, and DS samples decreased as with the increase in the immersion time (from 0 to 24 h) (Figure 3b), which reflected that the 3.5% NaCl solution could promote the dissolution of zinc-based coatings, possibly because of localized penetration of the electrolyte through the coatings. It is important to mention here that the GS coating is purely a metallic coating and that cold galvanized coatings are compared to metallic Zn particles homogeneously dispersed in the polymeric acrylic-based phase. The polymeric phase could certainly affect the dissolution tendency of the Zn particles that directly control the protection of the steel substrate. Shi et al. [30] explained that in the initial stage, the change in the semi-circle diameter was associated with the variation in the OCP values upon exposure to the electrolyte. The OCP of the MS sample did not vary appreciably during 24 h of exposure in 3.5% NaCl, as shown in Figure 2a, and no change in the semi-circle diameter was observed. The origin of a second time constant in the impedance spectra of Zn-based coatings exposed to the soil and seawater has also been explained by other researchers [28,31]. The GS sample immersed in 3.5% NaCl also presented a single time constant after one-hour exposure (Figure 3a). This suggested that there was no change in the dissolution mechanism of the GS compared to the MS samples and is simulated to the same EEC model, as shown in Figure 5a. However, after one-hour exposure to the 3.5% NaCl solution, the R_ct_ value of the GS sample (414.4 ohm·cm^2^) became lower compared to the MS sample (744.6 ohm·cm^2^). After 24 h of immersion, the *R*_ct_ of the GS sample decreased to 37.81 ohm·cm^2^. The origin of a small capacitive loop within the low-frequency regime corresponded to the specific adsorption of the electrolyte species on the surface (i.e., OH^−^, Cl^−^, and H_2_O), which could react with the highly active Zn to form corrosion products and is simulated as Y_ads_ in the EEC model, as shown in Figure 5b. 

The BS and DS coated samples presented two semi-circles and were simulated to the two-constants EEC models as shown in Figure 5c,d. These results also validated the composite structure of the coatings containing resistive polymeric phase and active Zn metallic phase. The coating resistance (*R*_c_) of the BS sample corresponding to the polymeric phase was found to be 21.35 ohm·cm^2^ after one hour of immersion, which remained almost similar (20.45 ohm·cm^2^) even after 24 h. The *R*_ct_ of the BS sample increased from 26.52 to 105.3 ohm·cm^2^ after 24 h. Initially, low *R*_ct_ indicated the accelerated dissolution of the active Zn-rich phase in the BS sacrificial coating. Further, an increase in the *R*_ct_ (after 24 h of exposure) was possibly attributed to the formation of corrosion products that restricted the further dissolution of the coating. For the DS coated sample, initially, the *R*_c_ was found to be relatively higher (95.61 ohm·cm^2^) than the BS coated sample but decreased appreciably to 32.25 ohm·cm^2^ after 24 h. Similarly, a significant decrease in the *R*_ct_ value (from 469.1 to 54.35 ohm·cm^2^) after 24 h was observed. This behavior corresponding to the accelerated dissolution of the DS coating. However, the initial higher *R*_c_ and *R*_ct_ of the DS coated sample compared to the BS sample highlighted the extended life of the sacrificial coating was related to the homogeneity in the coating thickness (that is difficult to achieve in brush coating) ensuring the effective protection of the steel substrate. In other words, the large *R*_c_ of the DS (compared to BS) obtained after 24 h of exposure also validated the improved barrier characteristics of the polymeric phase in this coating and is simulated to the EEC model shown in Figure 5d.

Figure 3c,d show the Bode plots of the coated samples after 1 and 24 h exposure in 3.5% NaCl, respectively. The Bode plots demonstrate the variation in the modulus of impedance |Z| and shift in phase angle with a change in the frequency of the applied AC potential signals. Under applied AC potential, the magnitude of the current response is associated with electrochemical processes occurring at the electrode/electrolyte interface as a function of frequency. No appreciable change in the MS Bode plots (both impedance and phase angle curve) was observed after 1 and 24 h of exposure. The GS phasor curves highlighted the phase angle >0° at high frequency, which corresponds to its inductive behavior. At low frequency, an appreciable decrease in the |Z| of the GS after 24 h (from 414 to 200.81 ohm·cm^2^) pointed out the decrease in charge transfer resistance. The |Z| of the BS was lower (302.1 ohm·cm^2^) than the |Z| of GS after 1 h of exposure but increased to ~405 ohm·cm^2^ after 24 h of exposure. No considerable change in the |Z| values was observed in the Bode plots of the DS sample. However, as a function of frequency, a noticeable change in the phase angle was observed after 1 and 24 h of exposure to the saline medium. 

The Nyquist plots of the MS and Zncoated samples exposed to the soil are shown in Figure 4a,b. The EEC model used to simulate the impedance spectrum of the MS sample is shown in Figure 5d. The significantly higher *R*_s_ (~173.4 ohm·cm^2^) of the soil compared to 3.5% NaCl were evident in these results. All MS and Zn-coated samples presented two-time constants in the soil, and the EEC model used to simulate the electrochemical processes is shown in Figure 5d. However, the BS sample registered the noticeably different characteristics in the low-frequency regime that is associated with the diffusion process and is indicated as a Warburg constant (W) in the EEC model (Figure 5e). For the MS sample, the *R*_c_ and *Y*_c_ in the EEC model were replaced with film resistance (*R*_f_) and film constant phase element (*Y*_f_), which is associated with the presence of a thin product layer on the surface of steel that could form in the soil as reported elsewhere [32]. The *R*_f_ of the MS sample was 581.7 ohm·cm^2^ after 1 h and slightly increased to 597.8 ohm·cm^2^ after 24 h, representing the growth of the product layer. On the other hand, during 24 h of exposure to the soil, a slight decrease in the *Y*_f_ (from 46.88 to 39.51 μS.s^nf^/cm^2^) was associated with the charge transport characteristics of the surface film, which was able to form at the steel surface in the moist soil. 

For the GS sample, the increase in *R*_c_ (from 87.42 to 215.7 ohm·cm^2^) with the increase in exposure time was observed. Similarly, the DS coated sample exhibited the highest R_c_ (453.5 ohm·cm^2^) compared to other coated samples that further increased to 471.5 ohm·cm^2^ after 24 h due to the fact that the slow dissolution of the coating could withstand longer and the barrier polymeric phase could restrict the ingress of aggressive ions and moisture in the soil towards the steel substrate. This coating could resist the rapid attack for an extended period and may effectively protect the steel substrate [33,34,35].

Compared to the DS sample, the BS coated sample depicted a different impedance trend in the soil and is somehow related to its relatively positive OCP (Figure 2b). This potential did not suffice to meet the requirement of cathodic protection as discussed above. The significantly low R_ct_ of the BS sample compared to the DS sample corresponded to the inhomogeneity in the coating. The *R*_ct_ value of the BS was low (about 44.1 ohm·cm^2^) after 1 h exposure and slightly increased to 78.9 ohm·cm^2^ after 24 h. The dissolution tendency of the cold galvanized coatings not only depends on the active Zn-rich phase but also on its dispersion and binding with the surface provided by the polymeric phase, which behaves as a physical barrier to protect steel substrate from corrosion. Figure 4c,d show the Bode plots of the coated samples after 1 and 24 h exposure in soil, respectively. The impedance curve of GS and MS exhibited almost similar trends after 1 and 24 h of exposure. However, a considerable increase in the |Z| of the BS coated sample was observed, which may be related to the additional resistance of the corrosion product that has possibly formed on the surface. This can also be estimated from the more negative shift in phase angle within an intermediate and low-frequency regime indicating the capacitive behavior of the product layer after 24 h of exposure in soil. On the other hand, the |Z| of the DS sample was appreciably high compared to the BS and GS coatings and remained unaffected even after 24 h of exposure. 

### 3.4. Cyclic Polarization

**Figure 6:**
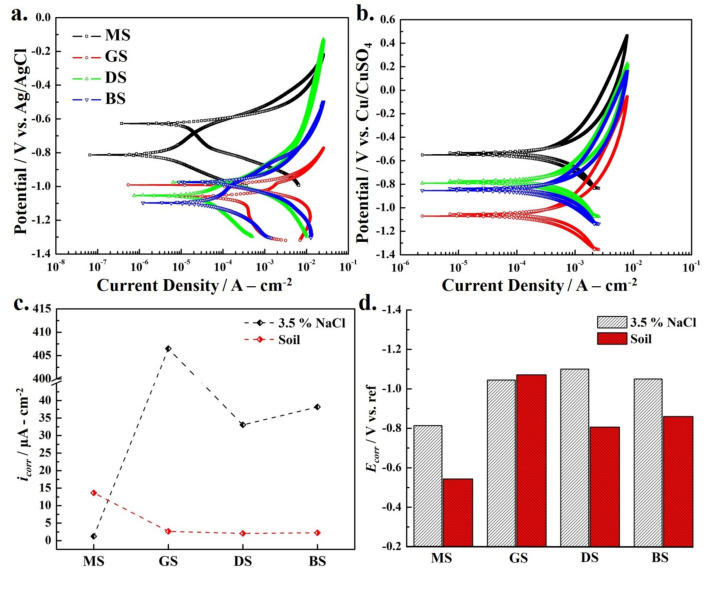
Cyclic Polarization (**a**) 3.5% NaCl environment (**b**) soil environment **(c)** corrosion current profile and (**d**) corrosion potential

The cyclic polarization method is used to assess the localized corrosion tendency of the active phase of the cold galvanized coatings in an aggressive aqueous saline solution and compared with the purely metallic Zn coating. During reverse polarization, the hysteresis loop is formed, which reflects the susceptibility of localized attack on the coated samples. The relatively large current response in the reverse anodic polarization scan (compared to the initial forward anodic current) is represented as positive hysteresis. If there is a negative hysteresis loop, or if the reverse scan curve essentially retraces the forward scan, the surface is considered to be least prone to localized dissolution. The area of the positive hysteresis loop corresponds to the overall preferential dissolution tendency and growth in the locally damaged area [12,36,37,38]. Figure 6a shows the comparison of cyclic polarization curves in saline solution after 24 h of immersion. The cyclic polarization curve of MS exhibited active dissolution of the steel surface and a small positive hysteresis loop which indicated (a large current during the reverse anodic scan) its uncontrolled dissolution tendency in saline solution. Similarly, the GS sample registered a large anodic current during the forward polarization scan representing its active dissolution. Compared to MS, in the coated samples (GS, DS, and BS), the rapid increase in anodic current at small anodic overpotential validated the active dissolution of the Zn-rich phase in 3.5% NaCl solution. In other words, compared to the MS sample, the very negative corrosion potential (*E*_corr_) and large anodic current produced by the GS and cold galvanized coatings (compared to MS) verified the rapid sacrificial dissolution of the Zn-rich phase in the coatings and their protection capability. During reverse anodic polarization of the coated samples, the curves retraced the forward anodic scans, thus validating their dissolution [36].

Figure 6b shows the comparison of cyclic polarization curves of uncoated and coated steel samples after 24 h of exposure to soil. The cyclic polarization curve of MS and coated samples showed a positive hysteresis loop in the soil depicting their uniform dissolution without having any signs of localized attack. The relatively large anodic polarization (large Tafel slope) of the MS and coated samples in the soil after 24 h of exposure is indicated by the small increase in current at large overpotentials. The DS sample also exhibited a relatively slow increase in current (8–20 mA·cm^−2^) at large overpotentials, which is possibly associated with the uniformity of the coating and well-controlled dissolution of the active Zn-rich phase.

The overall uniform corrosion tendency of the MS, GS, and ZRCG coated samples was also evaluated by linear fitting and extrapolation of the Tafel region of both anodic and cathodic polarization curves. The quantitative information about the dissolution kinetics of the MS, GS, and ZRCG coated samples (in both saline and soil environments) is given in Table 3. 

The negative corrosion potential (*E*_corr_) of GS and ZRCG coated samples compared to the MS sample in 3.5% NaCl and soil conditions confirmed the preferential dissolution tendency of the coating compared to the MS substrate. The value of E_corr_ for MS is about −813 mV vs. Ag/AgCl while GS, DS, and BS are −1045, −1052, and −1070 mV vs. Ag/AgCl in 3.5% NaCl, which supported the OCP results. The surface roughness of the GS sample was low (R_a_ = 0.45 μm) compared to DS (0.80 μm) and BS (1.15 μm) samples, which could be related to its relatively less negative *E*_corr_. It is therefore suggested that by changing the coating application method of cold galvanizing, the *E*_corr_ may be affected due to a change in surface roughness as it was shifted to more negative potential in the case of DS and BS samples. The surface roughness of the ZRCG coatings can be easily controlled via dip coating, and the process can be optimized effectively to ensure reproducibility. However, the manually applied brush coating process may vary because of many factors, i.e., coating layers, brush movement, direction, pressure, and applicator skills etc.

In a 3.5% NaCl solution, the corrosion current (*i*_corr_) of coated samples was found to be much higher compare to the MS sample. The value of *i*_corr_ for GS is about 406.5 μA·cm^−2^, which is approximately four hundred times greater than the value of MS (1.216 μA·cm^−2^), highlighting its enhanced dissolution rate (4.71 mm/year). The *i*_corr_ value confirmed that the zinc electroplated samples would present good protection to steel structures by their preferential dissolution. The *i*_corr_ values for DS and BS were 33.06 and 38.18 μA·cm^−2^, respectively, which were approximately 10 times smaller than GS. The possible reason for the low corrosion rate of cold galvanized DS and BS samples is due to the barrier character of the acrylic-based resin, with 96% zinc contents in the dry film compared to GS coating, which is purely metallic Zn. It is for this reason that DS (0.38 mm/year) and BS (0.44 mm/year) samples presented a small corrosion rate compared to the GS sample.

Figure 6c shows the comparison of *i*_corr_, whereas Figure 6d shows the comparison of E_corr_ in 3.5% NaCl and soil environments. The MS has a large *i*_corr_ value of 13.66 μA·cm^−2^ in soil environment compared to an *i*_corr_ of about 1.216 μA·cm^−2^ in 3.5% NaCl solution. Moreover, the *E*_corr_ value of MS in soil was relatively more positive (noble) (−543.6 mV vs. Cu/CuSO_4_) compared to −813 mV vs. Ag/AgCl as measured in the saline solution. The *E*_corr_ value for GS was found to be almost equal in both environments. For GS samples, the *i*_corr_ value increased to 406.5 μA·cm^−2^ in a saline solution. However, this sample presented low *i*_corr_ (2.64 μA·cm^−2^) in soil, possibly due to the formation of hydrated zinc oxide and/or chloride species on its surface, which may control the dissolution tendency of GS, suggesting its possible use to protect the steel structures buried in soil [27]. The values of *i*_corr_ for DS and BS in the saline environment were 38.18 and 33.06 μA·cm^−2^, which were much higher than in soil, therefore suggesting that cold galvanized coatings would preferentially dissolve in the 3.5% NaCl solution by protecting the steel substrate.

### 3.5. Salt Spray Testing

**Figure 7:**
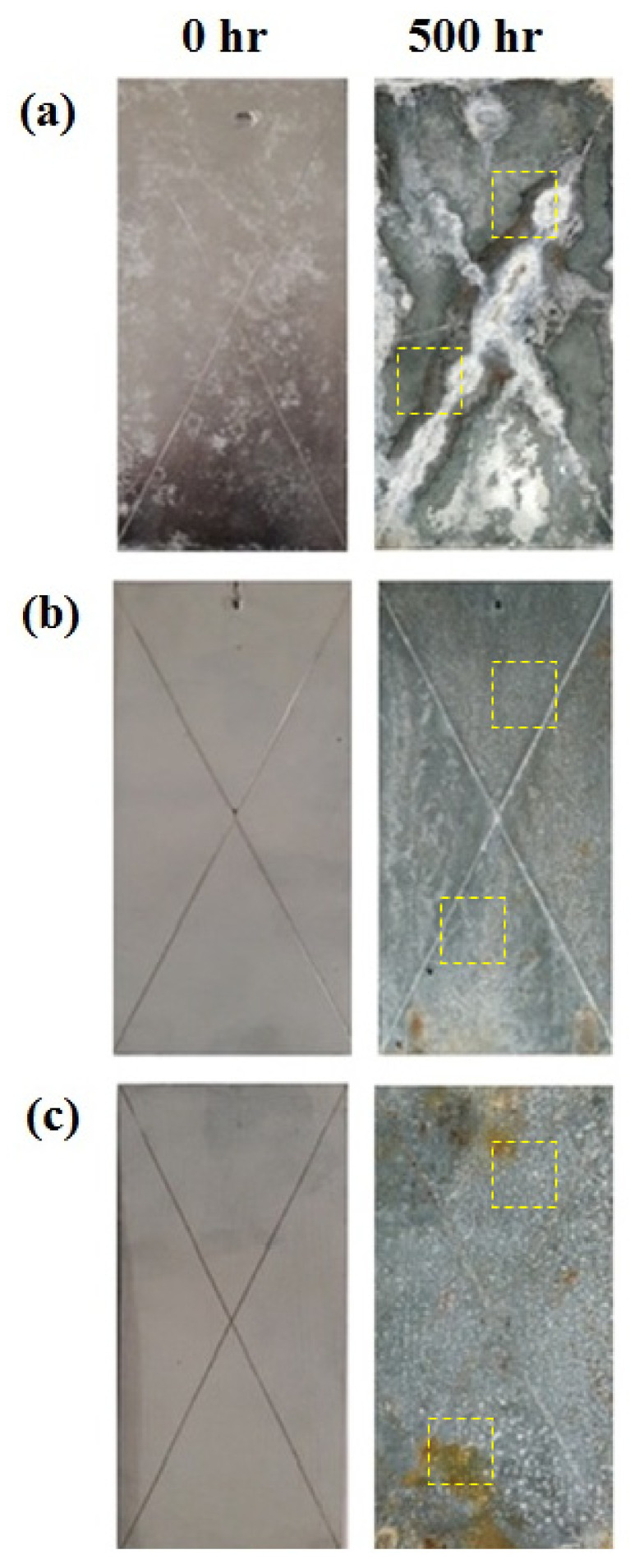
Surface features of coated panels after 500 h of exposure to salt spray: (**a**) GS, (**b**) BS, (**c**) DS.

The salt spray test is a commercially accepted qualitative approach to evaluate the performance of the coated samples in an aggressive environment. Figure 7 shows the coated panels before and after 500 h of exposure to the humid saline mist in a salt spray chamber. The formation of brown rust was not observed on GS and BS panels, whereas slight brown rust appeared on the DS panel. No blisters on coating panels were observed after 500 h of exposure, which reflected their good adhesion to the substrate. All the coated panels showed white rust after 500 h of exposure, which corresponds to the sacrificial dissolution of zinc and formation of Zn_5_(OH)_2_Cl_8_ corrosion product [39,40]. Many studies in the past have explained that corrosion of the galvanized steel occurs in three stages [41,42]. Initially, zinc dissolved into Zn^2+^ in the first stage, which is followed by the interaction of these ions with the OH^−^ species produced on the surface due to H_2_O reduction. The hydrated oxide corrosion products Zn(OH)_2_ (in absence of chloride) and Zn_5_(OH)_2_Cl_8_ (in presence of chloride) form as an adherent and a porous layer with a white color appearance [41,43,44]. As the salt spray panels have a cross-hatch, the zinc provides sacrificial protection to the steel by forming white rust on the scratches of the coatings [45,46,47], as shown in Figure 7. A large amount of white product formation on the GS sample corresponded to the preferential dissolution of the coating and provided an additional physical barrier to the electrolyte approaching the substrate, hence decreasing the corrosion of steel substrate in aggressive saline solution. The DS panels presented relatively clean surfaces (Figure 7b) even after exposure to salt spray, which indicated the effective galvanic protection of steel substrate compared to GS and BS coated panels.

## 4. Conclusions

The following conclusions are drawn from this research work:

- In contrast to electroplated Zn coating, the controlled dissolution tendency of the ZRCG samples and their larger stability in both 3.5% NaCl and soil environment was related to their composite structure.
- In a saline solution (3.5% NaCl), the Zn electroplated coating dissolves rapidly in an uncontrolled manner. On the other hand, the ZRCG coatings effectively protect steel substrate and meet the cathodic protection criteria (OCP −900 mV vs. Ag/AgCl). In soil, the BS sample the OCP was found to be >−850 mV vs. Cu/CuSO_4_, indicating its effectiveness towards cathodic protection of MS.
- Compared to ZRCG coated samples, the very high dissolution rate of the Zn-electroplated steel sample (4.72 mm/year) in 3.5% NaCl solution corresponded to its limited life and short term protection of the steel substrate. In other words, it is evaluated that the ZRCG coatings could last longer and their protection capacity is extended due to their composite structure.
- In saline soil, the ZRCG coating presented a relatively low dissolution rate (~0.025 mm/year) compared to ~0.44 mm/year in a 3.5% NaCl solution.
- From the impedance spectroscopy results, the quantitative estimation of the model parameters confirmed the decrease in charge transfer resistance of the coated samples after 24 h of immersion in both 3.5% NaCl and soil environments. This indicated the protection of steel substrate by the sacrificial dissolution of Zn coatings.
- During salt spray testing, a large amount of white corrosion product on the GS sample indicating the complete dissolution of the Zn coating, whereas the surface of the DS and BS samples was homogeneously dissolved with the origin of some rust spots.
- The DS sample exhibited better performance compared to the BS samples due to its controlled dissolution tendency, which is an effective and relatively improved protection capability of the steel substrate.
- Based on the experimental results, it is deduced that the polymeric phase in the ZRCG coatings not only assists the inter-particulate binding but also regulates the dissolution rate of the Zn phase. However, the consistency in the coating layer, control in coating thickness, and surface homogeneity strongly depend on the coating application method. Therefore, the better performance of the sacrificial coatings was achieved by dip coating, which is a procedure that is easy to optimize compared to the brush coating method.

## Figures and Tables

**Table 1:** Designation of the uncoated steel and coated samples.

Specification of Panels	Designation
Mild steel	MS
Zinc Electroplated	GS
ZRCG coating applied via brushing	BS
ZRCG coating applied via a dipping method	DS

**Table 2:** Electrochemical parameters obtained from the fitting of experiment impedance spectra.

Sample	*t* (hour)	*R*_s_ (ohm·cm^2^)	*Y*_c_ (mS.s^nc^/ cm^2^)	*n*_c_	*R*_c_ (ohm·cm^2^)	*Y*_dl_ (mS.s^ndl^/ cm^2^)	*n*_dl_	*R*_ct_ (ohm·cm^2^)	*L* (nH·cm^2^)
**3.5% NaCl**									
MS	1	0.97 ± 0.05	-	-	---	9.06 ± 0.01	0.71 ± 0.02	744.6 ± 3.4	-
	24	1.55 ± 0.08	-	-	-	8.55 ± 0.011	0.689 ± 0.002	693.4 ± 3.7	-
GS	1	1.31 ± 0.06	-	-	-	0.07 ± 0.01	0.83 ± 0.02	414.4 ± 2.6	-
	24	0.69 ± 0.03	0.624 ± 0.03	0.843 ± 0.02	-	124.4 ± 0.01	0.080 ± 0.02	37.81 ± 2.1	120.6 ± 2.7
BS	1	1.35 ± 0.07	1.39 ± 0.07	0.589 ± 0.02	21.35 ± 1.06	242.2 ± 0.02	0.684 ± 0.02	26.52 ± 1.7	-
	24	1.32 ± 0.07	4.24 ± 0.21	0.499 ± 0.01	20.45 ± 1.02	175.6 ± 0.02	0.703 ± 0.02	105.3 ± 3.4	-
DS	1	1.26 ± 0.06	117.9 ± 5.895	0.781 ± 0.01	95.61 ± 2.78	0.07 ± 0.03	0.56 ± 0.02	469.1 ± 2.1	-
	24	1.34 ± 0.07	181.4 ± 9.07	0.775 ± 0.01	32.25 ± 1.61	3.551 ± 0.01	0.470 ± 0.02	54.35 ± 1.7	-
**Soil**									
MS	1	173.4 ± 8.7	0.05 ± 0.02	0.23 ± 0.02	581.7 ± 5.17	3.58 ± 0.01	0.65 ± 0.02	894.5 ± 3.3	
	24	191.2 ± 9.56	0.04 ± 0.01	0.32 ± 0.02	597.8 ± 3.1	2.92 ± 0.01	0.51 ± 0.02	910.3 ± 2.8	
GS	1	45.01 ± 2.3	596.9 ± 9.84	0.492 ± 0.02	87.42 ± 4.1	0.21 ± 0.02	0.74 ± 0.02	330.8 ± 1.7	-
	24	59.9 ± 3.0	600.2 ± 8.21	0.46 ± 0.02	215.7 ± 2.7	0.32 ± 0.02	0.74 ± 0.02	274.6 ± 2.1	-
BS	1	123.7 ± 6.2	97.4 ± 4.4	0.319 ± 0.01	296.6 ± 3.6	10.41 ± 0.01	0.95 ± 0.02	44.1 ± 2.5	-
	24	134.6 ± 6.7	64.5 ± 3.3	0.218 ± 0.01	333.8 ± 3.1	24.5 ± 0.02	0.10 ± 0.02	78.9 ± 2.1	-
DS	1	117.6 ± 5.9	76.6 ± 3.82	0.38 ± 0.02	453.5 ± 3.6	3.48 ± 0.02	0.56 ± 0.02	381.3 ± 2.6	-
	24	121.0 ± 6.1	51.4 ± 2.6	0.37 ± 0.01	471.8 ± 3.0	3.31 ± 0.01	0.48 ± 0.02	805.7 ± 2.9	-

**Table 3:** Kinetic parameters of the MS, GS, and ZRCG coated samples exposed to saline and soil environments obtained from the polarization curves.

	*β*_a_ (mV·Decade^−1^)	*β*_c_ (mV·Decade^−1^)	*i*_corr_ (μA·cm^−2^)	*E*_corr_ (mV)	Dissolution Rate (mm/Year)
**Saline**					
**MS**	87.72 ± 3.15	96.00 ± 4.51	1.216 ± 2.15	−813 ± 3	0.014 ± 0.001
**GS**	88.31 ± 2.51	-	406.5 ± 3.75	−1045 ± 5	4.720 ± 0.015
**BS**	125.6 ± 4.58	124.4 ± 2.11	38.18 ± 2.51	−1070 ± 6	0.443 ± 0.004
**DS**	112.4 ± 1.95	214.9 ± 3.77	33.06 ± 4.11	−1052 ± 4	0.384 ± 0.003
**Soil**					
**MS**	132.05 ± 1.44	148.2 ± 2.1	13.66 ± 0.78	−543.6 ± 3	0.158 ± 0.002
**GS**	79.93 ± 2.31	155.8 ± 1.2	2.64 ± 0.91	−1071 ± 5	0.031 ± 0.003
**BS**	198.31 ± 3.24	151.4 ± 1.8	2.22 ± 0.77	−805.9 ± 2	0.026 ± 0.001
**DS**	151.24 ± 2.95	161.6 ± 1.7	2.07 ± 0.41	−859.7 ± 5	0.024 ± 0.004

Note: Dissolution rate is presented as corrosion rate and can be used interchangeably in this study.

## Data Availability

The data presented in this study are available on request from the corresponding author. The data are not publicly available due to privacy.

## Supplementary Materials

The following are available online at https://www.mdpi.com/1996-1944/14/4/744/s1, Figure S1: Schematic diagram of paint cell (a) 3.5 % NaCl (b) Saline soil. Table S1: Average roughness values of mild steel and coated panels, Table S2: Chemical composition of soil.
