# Supplementary material for: Sacrificial Dissolution of Zinc Electroplated and Cold Galvanized Coated Steel in Saline and Soil Environments: A Comparison
Source: Materials (Basel). 2021 Feb 5;14(4):744. doi: 10.3390/ma14040744 (PMC7915669; doi:10.3390/ma14040744)
Supplement: Supplementary file 1 [file materials-14-00744-s001.pdf]

materials MDPI

Supplementary

Sacrificial Dissolution of Zinc Electroplated and Cold Galvanized Coated Steel in Saline and Soil Environments: A Comparison

Ameeq Farooq [1,†], Umer Masood Chaudry [2,†], Ahsan Saleem [1], Kashif Mairaj Deen [1,3,*], kotiba Hamad [2,*] and Rafiq Ahmad [1,*]

[1] Corrosion Control Research Cell, Department of Metallurgy and Materials Engineering, University of the Punjab, Lahore 54590, Pakistan; ameeq.farooq@gmail.com (A.F.); ahsansaleem318@gmail.com (A.S.)
[2] School of Advanced Materials Science & Engineering, Sungkyunkwan University, Suwon 16419, Korea; umer@skku.edu
[3] Department of Materials Engineering, University of British Columbia, Vancouver, BC V6T 1Z4, Canada
* Correspondence: deen@mail.ubc.ca; (K.M.D.), hamad82@skku.edu (K.H.); drrafiqahmad@hotmail.com (R.A.)
† Authors contributed equally to this work.

Table S1. Average roughness values of mild steel and coated panels.

Sample	Average Roughness (R_a) (µm)
MS	4.12
GS	0.45
BS	1.15
DS	0.80

Table S2. Chemical composition of soil.

Description		Composition
pH of the soil paste		8.01
Sodium Adsorption Ratio (S.A.R)		5.3
% Saturation		41
Soluble Cations (mg/L)	Ca + Mg	3.5
	Na	7.0
	K	0
Soluble Anions (mg/L)	CO_3^-	-
	HCO_3^-	2.0
	Cl^-	1.25
	SO_4^-	7.25
% Sand (1–2 mm)		1.0
% Sand (0.05–1 mm)		5.7
% Silt		67.3
% Clay		26.0

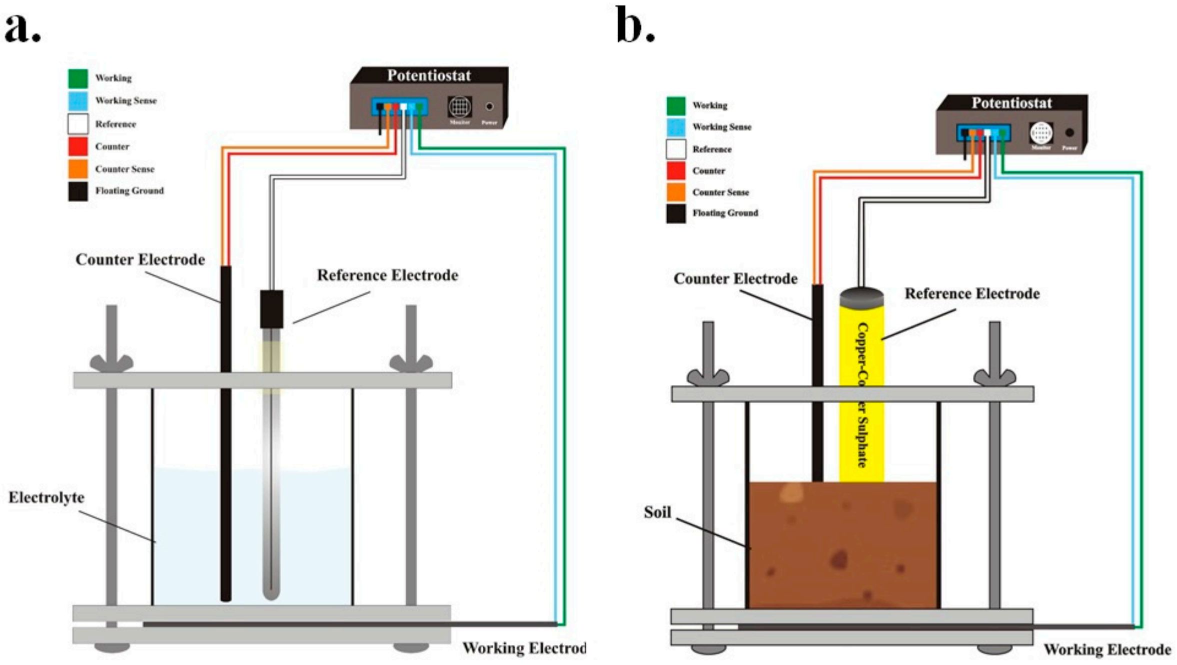

Figure S1. Schematic diagram of paint cell (**a**) 3.5 % NaCl (**b**) Saline soil.
